# *De novo* Transcriptome Reveals Gene Changes in the Development of the Endosperm Chalazal Haustorium in *Taxillus chinensis* (DC.) Danser

**DOI:** 10.1155/2020/7871918

**Published:** 2020-02-18

**Authors:** Shugen Wei, Lingyun Wan, Lili He, Ying Wei, Hairong Long, Xiaowen Ji, Jine Fu, Limei Pan

**Affiliations:** Guangxi Botanical Garden of Medicinal Plants, Nanning 530023, China

## Abstract

Loranthus (*Taxillus chinensis*) is a facultative, hemiparasite and stem parasitic plant that attacks other plants for living. Transcriptome sequencing and bioinformatics analysis were applied in this study to identify the gene expression profiles of fresh seeds (CK), baby (FB), and adult haustoria tissues (FD). We assembled 160,571 loranthus genes, of which 64,926, 35,417, and 47,249 were aligned to NR, GO, and KEGG pathway databases, respectively. We identified 14,295, 15,921, and 16,402 genes in CK, FB, and FD, respectively. We next identified 5,480 differentially expressed genes (DEGs) in the process, of which 258, 174, 81, and 94 were encoding ribosomal proteins (RP), transcription factors (TF), ubiquitin, and disease resistance proteins, respectively. Some DEGs were identified to be upregulated along with the haustoria development (e.g., 68 RP and 26 ubiquitin genes). Notably, 36 RP DEGs peak at FB; 10 ER, 5 WRKY, 6 bHLH, and 4 MYB TF genes upregulated only in FD. Further, we identified 4 out of 32 microRNA genes dysregulated in the loranthus haustoria development. This is the first haustoria transcriptome of loranthus, and our findings will improve our understanding of the molecular mechanism of haustoria.

## 1. Introduction

*Taxillus chinensis* (DC.) Danser, also called loranthus or “San Ji Sheng” (in Chinese), is a member of Loranthaceae family and mainly distributed in the southern and southwestern areas of China. It has a long history of being used in the Chinese traditional medicine, mainly because its stems and leaves can be used for the treatment of rheumatoid arthralgia, threat of abortion, and hypertension [1, 2]. Loranthus is a parasitic plant that attacks other plants, such as Aceraceae, Anacardiaceae, Euphorbiaceae, Fabaceae, Fagaceae, Juglandaceae, Moraceae, Rosaceae, and Rutaceae [2]. The successful parasitism is a key process for the plants to obtain water and nutrients from the host plants via specialized feeding structures called haustoria.

In plants, approximately 4,500 parasitic species belonging to 28 families, representing 1% of the dicotyledonous angiosperm species, have been reported [3]. Depending on the attachment site in the host plants, parasitic plants can be classified in to two groups—stem and root parasites. Also, according to the degree of host dependency, parasites can be facultative or obligate. Facultative parasites can live autotrophically but latter cannot, such as *Triphysaria* spp. and *Phtheirospermum* spp., while obligate parasites have to parasitize a host in order to complete their life cycles, for example, *Viscum* spp., *Cuscuta* spp., *Orobanche* spp., and *Striga* spp.. Further, parasitic plants can be classified as hemiparasites or holoparasites based on whether they have retained or completely lost the photosynthetic activity [3]. Based on these characteristics, loranthus is a facultative, hemiparasite, and stem parasite.

It has been reported that after seed germination, most parasitic plants will develop a functional haustoria depending on a second chemical signal also derived from the host exudate, such as 2,6-dimethoxy-p-benzoquinone (DMBQ), phenolic acids, and flavonoids (a haustoria-inducing factors (HIFs)) [4]. Some studies have shown the mechanisms of haustoria development in parasitic plants. For example, a single-electron reducing quinone oxidoreductase (TvPirin) is required to trigger the haustoria development in the roots of *Triphysaria versicolor* [5]. The seeds of *Santalum album*, an aggressive root hemiparasite, can germinate in sand or in vitro on Murashige and Skoog medium after a pretreatment of 2~8 mM GA_3_ for 12 h and then develop the haustoria within one month without the need for induction by HIFs [6–8]. Many is unknown about haustoria development in loranthus.

Transcriptome sequencing has been used to identify differentially expressed genes in the process of parasitism of *Cuscuta pentagona*, including genes encoding plant hormone (e.g., auxin, gibberellin, and strigolactones), transporters, and genes associated with cell wall modifications [9]. Also, it has been used to show that genes involved in cell wall metabolism, protein metabolism, and mitochondrial electron transport, genes related to auxin signaling and genes encoding nodulin-like proteins, were important for the haustoria development in *Santalum album* [4]. Transcriptome analysis also found that genes related to protein turnover, detoxification of reactive oxygen species, and fungal pathogenesis are abundant in the haustoria of *Golovinomyces orontii* [10]. Recently, small RNA sequencing characterized that some dodders' (*Cuscuta* spp.) microRNAs (miRNAs) could target the host (*Arabidopsis* thaliana) genes and further improve the parasitism [11].

In the present study, we constructed a transcriptome profile of haustoria development and identified genes encoding ribosomal proteins (RPs), transcription factors (TFs), ubiquitin, and disease-resistant proteins (DRPs) which might be involved in the loranthus haustoria development. Our results provide a valuable resource for further exploration and a basis towards understanding the molecular mechanisms of the haustoria development and underlying host-parasite interaction in angiosperms.

## 2. Materials and Methods

### 2.1. Plant Material

Fifty seeds of *Taxillus chinensis* (DC.) Danser were collected from the experimental field of Guangxi Botanical Garden of Medicinal Plants in China, confirmed by senior botanists and deposited in the herbarium of Guangxi Botanical Garden of Medical Plants (accession number: s0001794). Then, the seeds were peeled, washed with sterile water, placed on a germination dish, and incubated under the environment of 25°C temperature and 80% moisture. Every day, the seeds were lighted under 2000 Lx for 10 h. Three fresh seeds were collected as control (CK). After 10 days of incubation, three seeds with protruding seed-type radicle and tiny suction device were randomly collected (FB). After 20 days of incubation, the loranthus haustoria was formed and elongated, and the true leaves began to grow. Three of them were collected as adult haustoria (FD).

### 2.2. Total RNA Extraction and Transcriptome Sequencing

Total RNA was extracted using TRIzol reagent, as described [1, 12]. After the quality and quantity were determined by Agilent 2100 Bioanalyzer, total RNA (1 *μ*g) of each sample was used to construct the cDNA library using the TruSeq RNA Library Preparation Kit v2 protocol (Illumina), as described [13]. Then, cDNA libraries were quality controlled by the Agilent 2100 Bioanalyzer and qRT-PCR, followed by sequencing on the Illumina HiSeq2500 platform with paired-end 100 strategy.

### 2.3. *De Novo* of the Transcriptome

Raw data were cleaned using trim_galore (v0.5.0) and quality controlled using FASTQC (v0.11.7). Next, we used Trinity (v2.8.4) to *de novo* assemble the loranthus haustoria transcriptome with default parameters, as previously described [1].

### 2.4. Transcriptome Annotation

After the likely proteins were extracted from the assembled transcriptome using TransDecoder, they were annotated using Trinotate (v3.1.1). In this step, likely proteins were searched against the UniProtKB/Swiss-Prot database to identify known proteins, functional PFAM domains were identified using HMMER [14], signal peptides were predicted using SignalP [15], transmembrane domains were predicted using TMHMM Sever v2.0 [16], and rRNA transcripts were predicted using RNAMMER [17]. Then, EggNOG database (v4.1) [18] was searched against to identify proteins in EuKaryotic Orthologous Groups (KOG), Clusters of Orthologous Groups (COGs), and nonsupervised orthologous groups (NOGs).

Next, we annotated the assembled loranthus genes using KEGG pathway Gene Ontology (GO) databases. BLAST software was used to map the assembled genes to the NR database and the hits with and *e*-value of >1 × 10^−5^ were filtered. Remaining genes were processed to retrieve GO annotation in terms of biological process, cellular component, and molecular function by BLAST2GO [19]. Using the enzyme commission numbers produced by BLAST2GO, we mapped the assembled transcriptome to KEGG pathway database and obtained the pathway annotation.

### 2.5. Noncoding Gene and miRNA Annotation

Unannotated loranthus genes were processed by the Coding Potential Calculator (CPC, v2) with default parameters to identify potential long noncoding genes [20]. Then, all the plant mature microRNAs (miRNAs) were mapped to these noncoding genes to identify loranthus miRNAs using SOAP2 with maximal two mismatches [21]. Then, MIREAP was used to predict the miRNA precursor sequences, and psRobot was used to predict the target genes of miRNAs [22].

### 2.6. Gene Expression Profile and Differential Expression Analysis

Bowtie2 and RSEM tools were used to align clean reads to the assembled transcriptome and to profile the gene expression for each sample, respectively, [23]. Transcripts-per-million (TPM) reads method was for normalization, and lowly expressed genes (TPM < 5) were filtered. Then, differential expressed genes (DEGs) were identified using edgeR [24] with a strict criteria: log2 fold change (Log2FC) > 1 or <−1 and false discovery rate (FDR) of <0.05.

### 2.7. Functional Analysis

*p* value calculated using Fisher's exact test and *q* value calculated by the R package “qvalue” were used to identify enriched GO terms and KEGG pathways (*p* value of <0.05 and *q* value of <0.05). Human or other animal-related GO terms and pathways were filtered.

### 2.8. qRT-PCR

We randomly selected 9 genes for qRT-PCR validation, and 18S rRNA was used as internal control. Forward and reverse primers were predicted using Primer3 and synthesized at BGI-Shenzhen. The procedure of qRT-PCR experiment was same as our previous study [1]. The expression of genes was shown in ΔCt. ΔΔCt was used to present the difference of gene expression between two samples. Then, we used relative normalized expression (RNE) to show the gene expression changes: *RNE* = 2^−ΔΔCt^. *p* values were calculated using the multiple *t* tests function in Prism GraphPad 8.0.

## 3. Results

### 3.1. Plants, Sequencing, and *De Novo* Analysis

**Figure 1:**
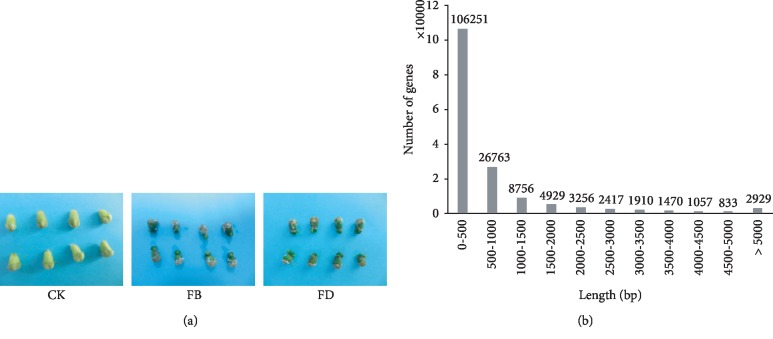
Loranthus seeds and Trinity *de novo* analysis. (a) Image captures of seeds in three conditions: fresh (CK), baby haustoria (FB), and adult haustoria (FD). (b) Length distribution of assembled loranthus haustoria genes.

Compared to CK, the green colors of FB and FD seeds were darker (Figure 1(a)). In addition, FB seeds produced seed-type radicle and tiny suction device. FD seeds formed and elongated the haustoria, and their true leaves began to grow. We generated a total of ~322.92 million reads (average: ~35.88 million reads) for these samples. After data cleaning, ~321.92 million reads were obtained, and Trinity assembled 160,571 loranthus genes that can produce 266,379 transcripts (Table 1). The size of the loranthus haustoria transcriptome was ~110 Mb, the GC percentage was 42.83%, the N50 was 1,191 bp, which revealed that 50% of the assembled loranthus genes were >1,191 bp, and the average gene length was 685.36 bp. Furthermore, gene length distribution (Figure 1(b)) showed 106,251 (66.17%) genes between 200 bp to 500 bp and 8,199 (5.11%) genes longer than 3000 bp.

### 3.2. Annotation of Coding and Noncoding Genes

**Figure 2:**
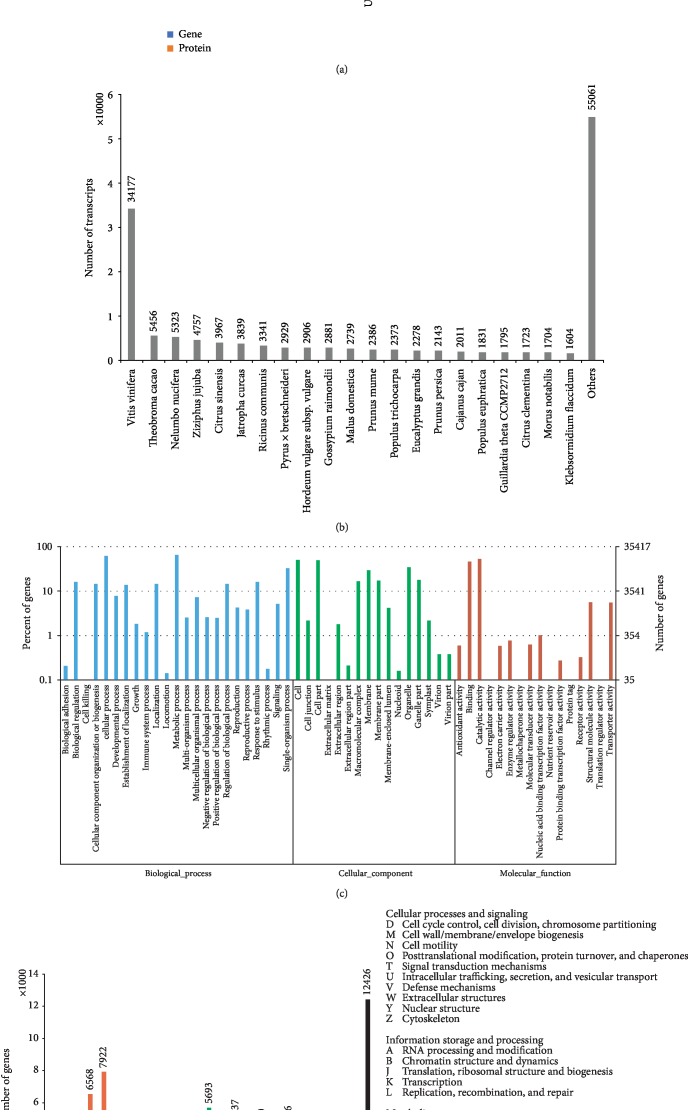
Annotation of the assembled transcriptome. (a) Number of genes aligned to databases. (b) Number of genes aligned to different species. (c) GO annotation of the assembled transcriptome. (d) COG annotation.

We next aligned the assembled genes to public databases, including NCBI nonredundant (NR), UniProt/SwissProt, GO, and KEGG pathway (Figure 2(a)**)**. It was shown that the 64,926 genes aligned to NR and that the top five species that are aligned by loranthus genes were *Vitis vinifera* (grape, 34,147 transcripts), *Theobroma cacao* (cacao tree, 5,456 transcripts), *Nelumbo nucifera* (lotus, 5,323 transcripts), *Ziziphus jujube* (jujube, 4,757 transcripts), and *Citrus sinensis* (orange, 3,967 transcripts) (Figure 2(b)). Top two GO terms involved by the assembled genes were “metabolic process” (23,049 genes) and “cellular process” (22,002 genes) (Figure 2(c)) while the top KEGG pathway involved with the loranthus genes was “metabolic pathway” (ko01100, 11,714 genes). Notably, we found 1,757 genes related to the pathway of “plant-pathogen interaction” (ko04626).

Then, TransDecoder predicted 96,665 proteins encoded by the loranthus genes and 78,196 (derived from 56,472 genes) were aligned to UniProtKB/SwiiProt database (Figure 2(a)). Next, we identified 65,702 functional Pfam domains, 5,986 signal peptides, and 20,910 transmembrane regions in the likely proteins using HMMER, SignalP, and TMHMM, respectively (Figure 2(a)). Next, we aligned the loranthus genes to EggNOG database and found that the top three categories were “signal transduction mechanisms” (7,922 genes), “post-translational modification, protein turnover, and chaperones” (6,568 genes), and “translation, ribosomal structure and biogenesis” (5,693 genes) (Figure 2(d)).

RNAMMER predicted 19 genes that can produce ribosomal RNAs in the assembled genes. Next, CPC identified 99,817 potential long noncoding genes in the unannotated genes, of which 32 were predicted to encode microRNAs from 19 microRNA families (Supplementary [Supplementary-material supplementary-material-1]). Interestingly, we found that 3,457 protein coding genes might be specific to loranthus according to the CPC label.

### 3.3. Gene Expression Profile and Differential Expression Analysis

After lowly expressed genes (TPM < 5) were filtered, we identified 14,295, 15,921, and 16,402 genes in CK, FB, and FD, respectively, and found 12,888 genes commonly detected in all three samples. Next, we performed DEG analysis to identify genes involved in the loranthus haustoria development. Using edgeR, we identified 3,749 and 4,139 DEGs in FB and FD, respectively, compared to CK (Supplementary [Supplementary-material supplementary-material-1]). Among these DEGs, 1,543 upregulated and 1,086 downregulated genes were common to FB and FD. Pathway analysis showed that metabolism and environmental adaptation pathways were common to FB and FD, “amino sugar and nucleotide sugar metabolism” (ko00520) specific to FB and “mineral absorption” (ko04978) was specific to FD. GO enrichment analysis identified that “regulation of flower development” (GO:0009909), “cell tip growth” (GO:0009932), and “glycerol ether metabolic process” (GO:0006662) were shared by FB and FD DEGs; the top three biological processes specific to FB were “protein phosphorylation” (83 genes, GO:0006468), “single-organism cellular process” (72 genes, GO:0044763) and “defense response” (33 genes, GO:0006952); and “cellular metabolic process” (39 genes, GO:0044237), “cellular macromolecule metabolic process” (26 genes, GO:0044260), and “proteolysis” (20 genes, GO:0006508) were the top three biological processes specific to FD.

### 3.4. Gene Family Analysis

We next analyzed several gene families differentially expressed during the loranthus haustoria development and formation (Supplementary [Supplementary-material supplementary-material-1]), such as ribosomal protein (RP), transcription factor (TF), ubiquitin, heat shock protein (HSP), auxin, and disease-resistant protein (DRP) (Table 2).

#### 3.4.1. Ribosomal Protein

**Figure 3:**
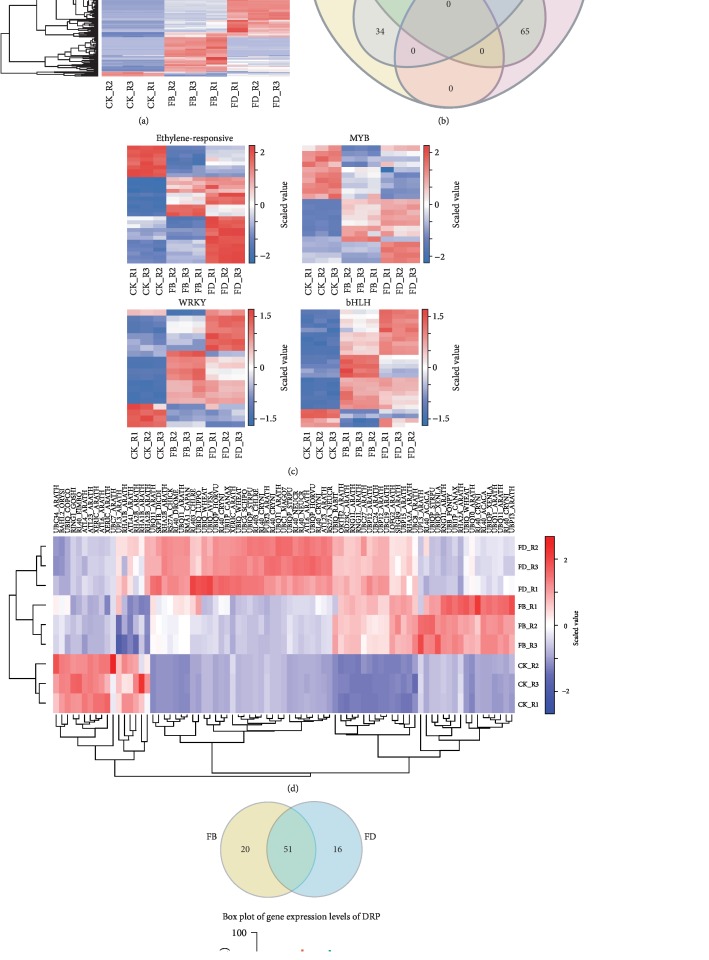
Gene family analysis of the DEGs. (a) Heat map of the RP gene expression. (b) Venn diagram of DEGs encoding TFs. (c) Heat maps of DEGs encoding different TFs. (d) Heat map of DEGs encoding ubiquitin proteins. (e) Comparison of DEGs encoding DRPs in FB and FD compared to CK.

Among the 2,576 RP genes, 255 were differentially expressed in the loranthus haustoria development (Figure 3(a)). In details, 7 and 5 RP genes (2 shared) were downregulated in FB and FD, respectively, compared to CK. Interestingly, CK 123 and 200 RP genes were upregulated in FB and FD, respectively, of which 79 were shared. Furthermore, out of the 121 upregulated RP genes exclusively in FD, 116 were downregulated in FB relative to FD. The expression profiles of RP genes in FB and FD indicate that they might have different functions in the development and formation of loranthus haustoria.

#### 3.4.2. Transcription Factor

We identified 863 TF genes in the loranthus haustoria, of which 174 were dysregulated in the developmental process. We found that most of the dysregulated TFs were shared (Figure 3(b)) by FB and FD. Next, we analyzed the expression changes of some TF subfamilies (Table 2, Figure 3(c)), including ethylene-responsive (ER), MYB, WRKY, and bHLH. Compared to CK, 9 ER, 5 MYB, 6 WRKY, and 11 bHLH TF genes were upregulated in both FB and FD (Figure 3(c)). Some TF genes were specifically upregulated in FB or FD. For example, 1 ER, 4 WRKY, and 5 bHLH TF genes were upregulated only in FB, while 10 ER, 5 WRKY, 6 bHLH, and 4 MYB TF genes were upregulated only in FD. This indicates that these TFs might be functionally translated as required for different stages. No TF genes were upregulated along with the loranthus haustoria development; however, we found some key TF genes started their upregulation from FB, including 9 ER, 4 WRKY, and 4 MYB (Supplementary [Supplementary-material supplementary-material-1]).

#### 3.4.3. Ubiquitin

We identified 1,194 ubiquitin genes in the loranthus haustoria, of which 81 were dysregulated (Table 2, Supplementary [Supplementary-material supplementary-material-1]). Among the 17 downregulated ubiquitin genes in FB relative to CK, 11 were also downregulated in FD and the other 6 were increased but had no significance in FD, compared to CK. While out of the 38 upregulated ubiquitin genes in FB compared to CK, 26 were upregulated in FD as well (Figure 3(d)). It is notable that 6 ubiquitin genes were increased along with the haustoria developmental process, including 3 polyubiquitin, 2 ubiquitin-40S RP, and 1 E3 ubiquitin-protein ligase genes.

#### 3.4.4. Disease Resistance Protein

We assembled 226 genes encoding DRPs in the loranthus haustoria, of which 94 were dysregulated (Table 2). It is notable that most of the DRP genes were upregulated.  Figure 3(e) reveals 87 (out of 94 DEGs) were upregulated in FB and FD, of which 51 were shared. We only identified 15 DEGs (7 upregulated and 8 downregulated) in FD relative to FB (Supplementary [Supplementary-material supplementary-material-1]). The upregulation of ubiquitin genes (Figure 3(e)) revealed that they might be functional in the loranthus haustoria development.

### 3.5. miRNA Host Genes and Their Target Genes

**Figure 4:**
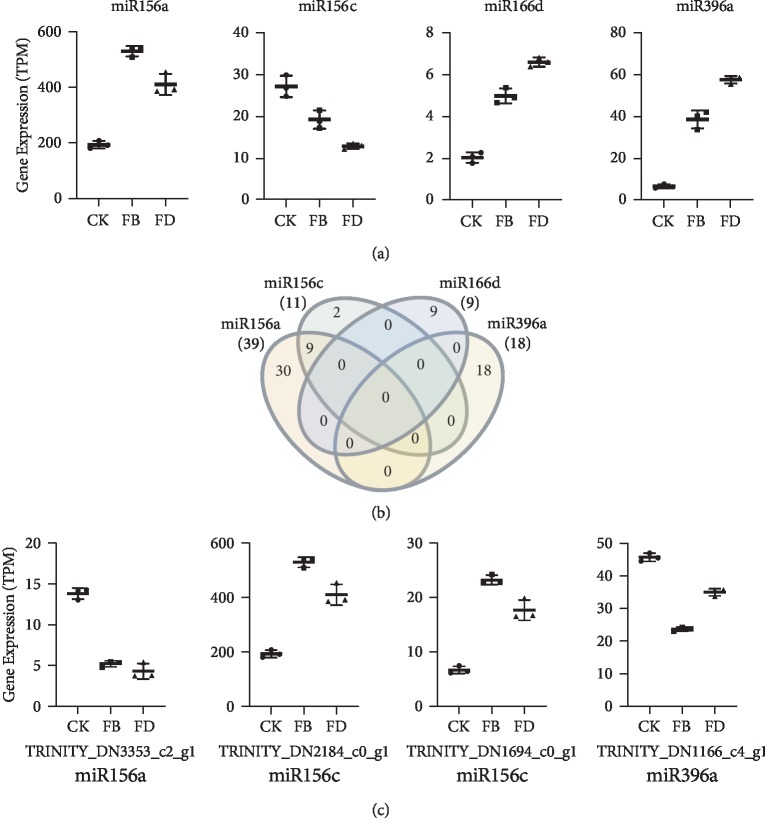
Expression of miRNA host genes and their targets. (a) Expression of host genes of miR156a, miR156c, miR166d, and miR396a. (b) Comparison of target genes for the four miRNAs. (c) Expression of target genes corresponding to miR156a, miR156c, miR166d, and miR396a.

We identified 32 miRNA host genes (Supplementary [Supplementary-material supplementary-material-1]) and 4 (miR156c, miR156d, miR166d, and miR396a) were dysregulated in the process (Figure 4(a)). We next predicted the target genes for these miRNAs and found that they had no common target genes except miR156a and miR156c (Figure 4(b)). The dysregulation of miRNA host genes might explain the change of their target genes, such as TRINITY_DN3353_c2_g1, TRINITY_DN2184_c0_g1, TRINITY_DN1694_c0_g1 (squamosa promoter-binding-like protein 2), and TRINITY_DN1166_c4_g1 (Figure 4(c)).

### 3.6. qRT-PCR Validation

We used qRT-PCR to validate the expression changes of 9 randomly selected genes in the loranthus haustoria development, and 18S rRNA was used as internal control. The primer sequences of these genes can be found in Supplementary [Supplementary-material supplementary-material-1]. The comparison of RNA-Seq and qRT-PCR results can be found in Table 3. Overall, 22 (81.48%) out of 27 events were agreed by both RNA-Seq and qRT-PCR. The expression patterns of 6 genes, including TRINITY_DN10066_c0_g1, TRINITY_DN3842_c0_g2, TRINITY_DN6353_c3_g1, TRINITY_DN6903_c0_g1, TRINITY_DN7338_c0_g1, and TRINITY_DN759_c0_g2, were consistent in RNA-Seq and qRT-PCR. High agreement of gene expression patterns in RNA-Seq and qRT-PCR indicates that the genes identified in this study might be functional during the loranthus haustoria development, which requires future functional experiments.

## 4. Discussion

Some studies have shown TFs' function in both parasitic plants and their hosts during the infection. For example, the upregulation of AtWRKY is important for the seeding site establishment of plant-parasitic nematodes [25]. Nearly one-half of the mobile mRNAs transferred from tomato or pumpkin to their parasitic plant *Cuscuta pentagona* were regulatory genes such as TFs and calmodulin proteins [26]. These evidences suggest that endogenous or exogenous TFs are important for the interaction of parasitic plants. We identified the dysregulation of bHLH, ER, MYB, and WRKY TFs (Table 2,Figure 3(c)), which may function in the formation and development of endosperm chalazal haustorium in *Taxillus chinensis*. These TFs have been reported to be inducible by the various environmental stresses, such as cold, drought, pathogen infection, and wounding, and be functional in the plant defense [27].

In parasitic plants, RP genes might play a key role in the survival and development. During the evolutional of *Epifagus virginiana*, although some RP genes are deleted, the *E. virginiana* plastid genomes are still transcribed and translated due to the fulfilled function by the nuclear components [28]. In addition, RPs have shown higher level of accumulation in resistant sunflower plants after the sunflower broomrape infection [29]. We found 258 out of 2,576 RP genes differentially expressed during the loranthus haustoria developmental and most are upregulated (Figure 3(a), Supplementary [Supplementary-material supplementary-material-1]). We assume that both host and parasitic plants have RP genes elevated during the early phase of parasitism.

Some studies have uncovered the functions of ubiquitin proteins in the parasitism in plants and animals. For example, a unique ubiquitin carboxyl extension protein (grUBCEP12) is secreted by the plant-parasitic nematode *Globodera rostochiensis* can promote successful plant parasitism through suppressing the plant's defense through the suppression of plant immunity and can further generate within root tissue the feeding cells essential for nematode development [30]. Rhiannon reported that E2 and E3 ubiquitin proteins secreted by the parasitic nematode *Trichinella spiralis* have the capacity of modifying the host skeletal muscle cells [31]. In this study, we identified 66, 176, and 540 genes encoding E1, E2, and E3 ubiquitin enzymes, respectively. Among them, 81 were differentially expressed (Table 2, Figure 3(d)), including 8 E2 and 29 E3 ubiquitin genes (Supplementary [Supplementary-material supplementary-material-1]). Based on these evidences, we assume that the secretion of ubiquitin genes and proteins by loranthus has positive efforts in the parasitism. While further experiments are required to study the functions of ubiquitin genes and proteins in the parasitism of loranthus.

A recent study reported that *Arabidopsis thaliana* mRNAs are targeted by miRNAs produced by *Cuscuta campestris* during the parasitism, resulting in mRNA cleavage, secondary siRNA production, and decreased mRNA accumulation [11]. Here, we predicted 32 miRNA host genes in the loranthus haustoria (Supplementary [Supplementary-material supplementary-material-1]) and identified the dysregulation of miR156c, miR156d, miR166d, and miR396a (Figure 4). Due to the limited information of loranthus genes, we only found a few genes targeted by these four miRNAs (Figure 4). Further experiments are required to identify the mature miRNA sequences and their function in the loranthus haustoria development.

## 5. Conclusions

In conclusion, we studied the transcriptome profiles of the loranthus haustoria development. We assembled 160,571 loranthus genes and annotated them by aligning them to NR, GO, KEGG, UniProt/Swiss-Prot, Pfam, and EggNOG databases. After lowly expressed genes were filtered, we identified 18,360 genes in the loranthus haustoria, of which 3,749 and 4,139 were dysregulated in FB and FD, respectively, compared to CK. Some important gene families were found to be related to the loranthus haustoria development, such as transcription factor, ubiquitin, ribosomal protein, and disease-resistant protein. Further, 32 miRNA host genes were identified and the dysregulation of 4 miRNA host genes might be one of the reasons for some genes which are dysregulated as well in the process. This is the first time to report the transcriptome of loranthus haustoria. It will provide valuable resources to other studies. More importantly, the findings of this study will improve our understanding of parasitism and contribute to the breeding program of loranthus.

## Figures and Tables

**Table 1:** Overview of the transcriptome sequencing and de novo analysis.

Sample	CK_R1	CK_R2	CK_R3	FB_R1	FB_R2	FB_R3	FD_R1	FD_R2	FD_R3
Raw_reads	24753420	43182897	15529733	34554423	50662597	32924448	42843339	44541750	33923755
Clean_reads	24663725	43043674	15411513	34379375	50514766	32915342	42671084	44404033	33913639
Genes	160571								
Transcripts	266379								
GC (%)	42.83								
N50 (bp)	1191								
Average_gene length (bp)	685.36								
Expressed_genes	13303	13428	13538	15307	15047	15087	15893	15564	15587

**Table 2:** Number of DEGs from different families identified in this study.

Gene_family	FB_vs_CK	FD_vs_CK	FD_vs_FB
Ribosomal protein	123/7	200/5	184/38
TF	80/46	101/41	32/12
*TF_bHLH*	15/4	17/2	1/3
*TF_ER*	9/10	18/7	13/1
*TF_MYB*	7/7	12/8	5/2
*TF_WRKY*	10/5	11/3	5/1
Ubiquitin	38/17	48/11	27/15
Disease-resistant protein	71/6	67/1	7/8

**Table 3:** qRT-PCR validation.

Gene_id	FB_vs_CK				FD_vs_CK				FD_vs_FB				UniProtID	Description
	Log2FC	*p* value	RNE^a^	*p* value	Log2FC	*p* value	RNE	*p* value	Log2FC	*p* value	RNE	*p* value		
TRINITY_DN10066_c0_g1	10.61	8.82E-29	1.54	0.0058	12.56	5.01E-44	2.21	0.0002	1.90	1.65E-05	1.44	0.0145	RL37A_OSTOS	60S ribosomal protein L37a
TRINITY_DN12554_c1_g1	0.00	1	-1.27	0.0178	10.64	2.34E-28	1.27	0.0014	11.01	2.28E-36	1.61	0.0005	RL403_CHLRE	Ubiquitin-60S ribosomal protein L40
TRINITY_DN2024_c3_g1	1.43	0.0009	1.25	0.0864	1.36	0.0017	1.52	0.0409	-0.08	1	1.21	0.1490	WRKY4_ARATH	Probable WRKY transcription factor 4
TRINITY_DN2307_c2_g1	-0.12	0.8821	1.33	0.0229	1.03	0.0236	-1.02	0.9803	1.14	0.0225	-1.36	0.2752	RAP24_ARATH	Ethylene-responsive transcription factor RAP2-4
TRINITY_DN3842_c0_g2	12.76	2.04E-45	1.31	0.2051	14.19	1.04E-57	2.59	0.0268	1.38	0.0031	1.97	0.0977	EF1A_PODCU	Elongation factor 1-alpha
TRINITY_DN6353_c3_g1	3.87	1.94E-17	1.40	0.1370	1.23	0.0134	1.04	0.5128	-2.65	1.04E-09	-1.35	0.2620	DRL21_ARATH	Putative disease resistance protein At3g14460
TRINITY_DN6903_c0_g1	-3.23	3.88E-14	-1.04	0.7034	-3.57	1.18E-16	-1.04	0.7243	-0.36	1	1.00	0.9948	AIL7_ARATH	AP2-like ethylene-responsive transcription factor AIL7
TRINITY_DN7338_c0_g1	0.18	0.8210	1.72	0.0045	1.51	0.0004	1.90	0.0017	1.32	0.0052	1.11	0.2517	ERF78_ARATH	Ethylene-responsive transcription factor 4
TRINITY_DN759_c0_g2	10.89	4.42E-30	1.09	0.2903	13.84	1.16E-54	1.98	0.0075	2.90	1.55E-11	1.81	0.0210	EF3_CANAL	Elongation factor 3

^a^RNE < 0 represents the downregulation.

## Data Availability

The raw sequencing data can be accessed from the NCBI Sequence Read Archive (SRA) platform (https://trace.ncbi.nlm.nih.gov/Traces/sra/) under the accession number SRA896707. The assembled transcriptome of loranthus haustoria can be accessed in the TSA database of NCBI under the accession number GHNL00000000.

## Supplementary Materials

Supplementary 1 Table S1. miRNA host genes and sequences.

Supplementary 2 Table S2: dysregulated genes identified in FB and FD compared to CK.

Supplementary 3 Table S3: forward and reverse primers used in the qRT-PCR experiment.

## References

[1] Wei S., Ma X., Pan L. (2017). Transcriptome analysis of Taxillusi chinensis (DC.) Danser seeds in response to water loss. *PLoS One*.

[2] Liu C. Y., Lin Y. C., Deng J. S., Liao J. C., Peng W. H., Huang G. J. (2012). Antioxidant, anti-inflammatory, and antiproliferative activities of Taxillus sutchuenensis. *The American Journal of Chinese Medicine*.

[3] Yoshida S., Cui S., Ichihashi Y., Shirasu K. (2016). The Haustorium, a specialized invasive organ in parasitic plants. *Annual Review of Plant Biology*.

[4] Zhang X., Berkowitz O., Teixeira da Silva J. A. (2015). RNA-Seq analysis identifies key genes associated with haustorial development in the root hemiparasite *Santalum album*. *Frontiers in Plant Science*.

[5] Bandaranayake P. C., Filappova T., Tomilov A. (2010). A single-electron reducing quinone oxidoreductase is necessary to induce haustorium development in the root parasitic plant *Triphysaria*. *Plant Cell*.

[6] Zhang X., Teixeira da Silva J. A., Duan J., Deng R., Xu X., Ma G. (2012). Endogenous hormone levels and anatomical characters of haustoria in *Santalum album* L. seedlings before and after attachment to the host. *Journal of Plant Physiology*.

[7] Nikam T. D., Barmukh R. B. (2009). GA3 enhances *in vitro* seed germination in *Santalum album*. *Seed Science and Technology*.

[8] Barrett D., Fox J. E. (1997). *Santalum album*: kernel composition, morphological and nutrient characteristics of pre-parasitic seedlings under various nutrient regimes. *Annals of Botany*.

[9] Ranjan A., Ichihashi Y., Farhi M. (2014). De novo assembly and characterization of the transcriptome of the parasitic weed dodder identifies genes associated with plant parasitism. *Plant Physiology*.

[10] Weßling R., Schmidt S. M., Micali C. O. (2012). Transcriptome analysis of enriched *Golovinomyces orontii* haustoria by deep 454 pyrosequencing. *Fungal Genetics and Biology*.

[11] Shahid S., Kim G., Johnson N. R. (2018). MicroRNAs from the parasitic plant *Cuscuta campestris* target host messenger RNAs. *Nature*.

[12] Chen M., Xu R., Rai A. (2019). Distinct shed microvesicle and exosome microRNA signatures reveal diagnostic markers for colorectal cancer. *PLoS One*.

[13] Chen M., Xu R., Ji H. (2016). Transcriptome and long noncoding RNA sequencing of three extracellular vesicle subtypes released from the human colon cancer LIM1863 cell line. *Scientific Reports*.

[14] Finn R. D., Clements J., Eddy S. R. (2011). HMMER web server: interactive sequence similarity searching. *Nucleic Acids Research*.

[15] Petersen T. N., Brunak S., von Heijne G., Nielsen H. (2011). SignalP 4.0: discriminating signal peptides from transmembrane regions. *Nature Methods*.

[16] Krogh A., Larsson B., von Heijne G., Sonnhammer E. L. L. (2001). Predicting transmembrane protein topology with a hidden markov model: application to complete genomes^1^. *Journal of Molecular Biology*.

[17] Lagesen K., Hallin P., Rødland E. A., Staerfeldt H. H., Rognes T., Ussery D. W. (2007). RNAmmer: consistent and rapid annotation of ribosomal RNA genes. *Nucleic Acids Research*.

[18] Powell S., Forslund K., Szklarczyk D. (2014). eggNOG v4.0: nested orthology inference across 3686 organisms. *Nucleic Acids Research*.

[19] Conesa A., Götz S., García-Gómez J. M., Terol J., Talón M., Robles M. (2005). Blast2GO: a universal tool for annotation, visualization and analysis in functional genomics research. *Bioinformatics*.

[20] Kong L., Zhang Y., Ye Z. Q. (2007). CPC: assess the protein-coding potential of transcripts using sequence features and support vector machine. *Nucleic Acids Research*.

[21] Li R., Yu C., Li Y. (2009). SOAP2: an improved ultrafast tool for short read alignment. *Bioinformatics*.

[22] Wu H. J., Ma Y. K., Chen T., Wang M., Wang X. J. (2012). PsRobot: a web-based plant small RNA meta-analysis toolbox. *Nucleic Acids Research*.

[23] Li B., Dewey C. N. (2011). RSEM: accurate transcript quantification from RNA-Seq data with or without a reference genome. *BMC Bioinformatics*.

[24] Robinson M. D., McCarthy D. J., Smyth G. K. (2010). edgeR: a bioconductor package for differential expression analysis of digital gene expression data. *Bioinformatics*.

[25] Grunewald W., Karimi M., Wieczorek K. (2008). A role for AtWRKY23 in feeding site establishment of plant-parasitic nematodes. *Plant Physiology*.

[26] Westwood J. H., Roney J. K., Khatibi P. A., Stromberg V. K. (2009). RNA translocation between parasitic plants and their hosts. *Pest Management Science*.

[27] Singh K., Foley R. C., Onate-Sanchez L. (2002). Transcription factors in plant defense and stress responses. *Current Opinion in Plant Biology*.

[28] Yoder J. I. (1999). Parasitic plant responses to host plant signals: a model for subterranean plant-plant interactions. *Current Opinion in Plant Biology*.

[29] Yang C., Xu L., Zhang N. (2017). iTRAQ-based proteomics of sunflower cultivars differing in resistance to parasitic weed *Orobanche cumana*. *Proteomics*.

[30] Chen S., Chronis D., Wang X. (2013). The novel GrCEP12 peptide from the plant-parasitic nematode *Globodera rostochiensis* suppresses flg22-mediated PTI. *Plant Signaling & Behavior*.

[31] White R. R., Ponsford A. H., Weekes M. P. (2016). Ubiquitin-dependent modification of skeletal muscle by the parasitic nematode, *Trichinella spiralis*. *PLoS Pathogens*.

